# Evaluation of Dye Compounds’ Decolorization Capacity of Selected *H. haematococca* and *T. harzianum* Strains by Principal Component Analysis (PCA)

**DOI:** 10.1007/s11270-015-2473-8

**Published:** 2015-07-01

**Authors:** Kamila Rybczyńska, Teresa Korniłłowicz-Kowalska

**Affiliations:** Department of Environmental Microbiology, Laboratory of Mycology, The University of Life Sciences, ul. Leszczyńskiego 7, 20-069 Lublin, Poland

**Keywords:** *H. haematococca*, *T. harzianum*, Anthraquinone dyes, Post-industrial lignin, Biodecolorization, Peroxidases

## Abstract

The selected strains of microscopic fungi, *Haematonectria haematococca* (BwIII43, K37) and *Trichoderma harzianum* (BsIII33), decolorized the following monoathraquinone dyes with different efficiency: 0.03 % Alizarin Blue Black B, 0.01 % Carminic Acid, 0.01 % Poly R-478, and 0.2 % post-industrial lignin. The most effective was the removal of 0.03 % Alizarin Blue Black B (50–60 %) and 0.01 % Carminic Acid (55–85 %). The principal component analysis (PCA) method was applied to determine the main enzyme responsible for the biodecolorization process of the dye substrates and indicated that horseradish-type (HRP-like), lignin (LiP), and manganese-dependent (MnP) peroxidases were responsible for the decolorization of anthraquinone dyes by the strains tested. The participation of particular enzymes in the decolorization of monoanthraquinone dyes ranged from 44.48 to 51.70 % for 0.01 % Carminic Acid and from 38.46 to 61.12 % for Poly R-478. The highest precipitation in decolorization of these dyes showed HRP-like peroxidase, respectively, 54–74 and 70–95 %. The degree of decolorization of 0.2 % post-industrial lignin by the selected strains of *H. haematococca* and *T. harzianum* amounted to 58.20, 61.38, and 65.13 %, respectively. The rate of 0.2 % post-industrial lignin decolorization was conditioned by the activity of HRP-like (71–90 %) and LiP (87–94 %) peroxidases.

**Electronic supplementary material:**

The online version of this article (doi:10.1007/s11270-015-2473-8) contains supplementary material, which is available to authorized users.

## Introduction

Filamentous fungi are the most promising among the pool of potential microorganisms important in decolorization and bioremediation processes. In comparison with prokaryotic microorganisms, filamentous fungi demonstrate certain advantages in biological decontamination. These are much greater surface contact with the dye substrate and unique physiological characteristics that allow decolorization of high concentrations of dye compounds and their use in the actual textile dye decolorization (Kaushik and Malik 2009; Ali 2010). Filamentous fungi are characterized by the ability to synthesize and secrete enzymes, organic acids, and other metabolites, which allow them to grow in a wide range of environmental pH (Mannan et al. 2005).

The best characterized strains among white rot basidiomycetes in terms of ability to decolorize dye substrates present in industrial wastewater are *Phanerochaete chrysosporium*, *Bjerkandera adusta*, *Trametes versicolor*, and *Pleurotus ostreatus* (Swamy and Ramsay 1999; Palmieri et al. 2005; Korniłłowicz–Kowalska et al. 2006; Eichlerová et al. 2007).

In recent years, the body of data on the decolorization capacities of microscopic fungi has been increasing. Similarly to the white rot fungi, microscopic fungi can degrade a broad spectrum of aromatic compounds. Some micromycete species of the genus *Fusarium*, *Aspergillus*, *Penicillium*, and *Trichoderma* degrade PAHs including anthracene, a precursor of synthetic dyes (Wu et al. 2010; Wu and Nian 2014), and purify industrial wastewater from olive oil (Robles et al. 2000), cotton delignification effluent (Souza et al. 2005), structurally diverse industrial dyes (Mannan et al. 2005; Shedbalkar et al. 2008; Anastasi et al. 2009; More et al. 2010), and kraft lignin (I alkali fraction) (Lopez et al. 2007; Yang et al. 2011).

Due to the scale of the problem, new microorganisms that would exhibit physiological properties of potential use in biodecolorization are continuously searched. In comparison to the white rot fungus, ligninolytic properties and potential practical use of the microscopic fungi are still not thoroughly investigated.

The aim of our study was to comprehensively evaluate decolorization ability of newly selected strains of microscopic fungi towards the aromatic dye compounds (anthraquinone derivatives) using principal component analysis (PCA) and identify the main enzymes (extracellular oxidoreductases) responsible for the decolorization of anthraquinone dyes and post-industrial lignin by selected microscopic fungi. The PCA method has been previously applied in studies on the assessment of the degree of decolorization and biodegradation of aromatic substrates by fungi (Ferraz et al. 1998; Lyra et al. 2009; Feng et al. 2011). Application of PCA not only allows to identify the main oxidoreductases responsible for the decolorization of particular dye substrates but also to determine the relationships between these enzymes and capture the differences in biodecolorization mechanism of structurally diverse anthraquinone derivatives. In addition to the information on the strength and direction of the correlation between the analyzed parameters, PCA method also defines the degree of involvement of different enzymes in the decolorization of dye substrates investigated.

## Materials and Methods

### Strain Sources

The strains of microfungi were isolated from cultivated soil (black earth, Phaeozemes according (acc.) to FAO) of the following composition (%): humus 3.61, N_og_ 0.290, and pH_KCl_ = 7.30 (southeastern Poland) and mature compost from lignocellulosic wastes (25.53 % pine bark, 10.63 % wheat straw, 51.06 % sawdust, and 12.76 % chicken feathers as a source of nitrogen) containing in total (g/kg dry matter) C_org_ = 498.7 and N_tot_ = 19.44).

### Dye Compounds

**Fig. 1:**
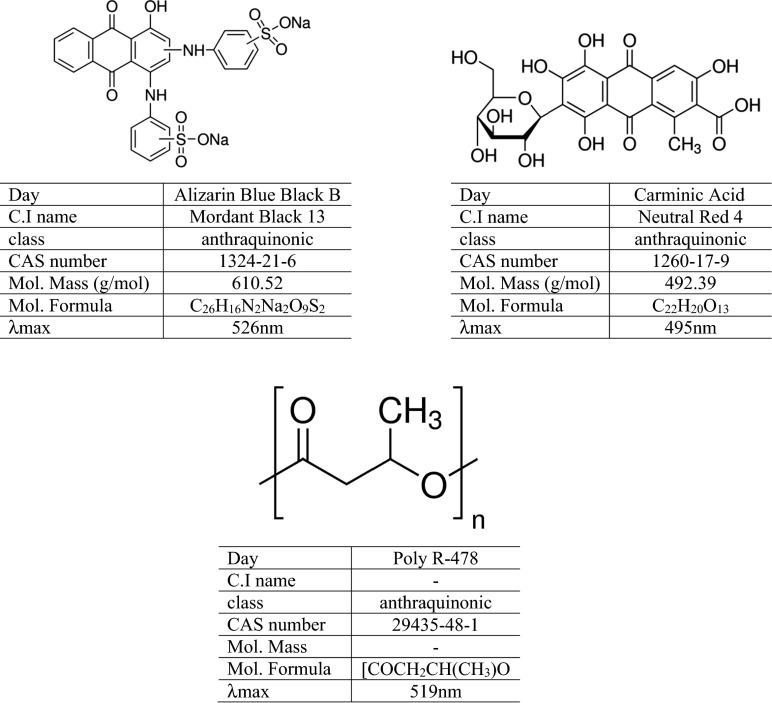
Dye structures

The study used the following monoanthraquinone dyes: Carminic Acid, Alizarin Blue Black B (Sigma) (Fig. 1), and textile blue used to dye textiles (Alizarin dye) obtained from the Institute of Dyes (Łódz, Poland) as well as polyanthraquinone dye Poly R-478 (Sigma) (Fig. 1).

The lignin waste was obtained from the StoraEnso (Ostrołęka, Poland), as a solid material precipitated from the first alkaline fraction with concentrated H_2_SO_4_. Prior to use, the lignin was dissolved in 0.1 M NaOH. The basic chemical properties of that fraction were as follows (g/kg dry matter): 408.2 carbon, 40.4 hydrogen, and 0.2 nitrogen.

### Fungal Isolation

Isolation of fungi from the soil was conducted using a post-industrial lignin as a substrate. Lignin (10 g) in polyamide-6 bags (5 × 5 × 6 cm, pores of 0.5 mm) was introduced to glass vessels with a volume of 1,000 cm^3^ filled with soil to a depth of ca. 5 cm from the soil surface. After adjusting the soil moisture to the level of 50 %, the height of soil layer was ca. 25 cm. Incubation was conducted at room temperature 20 ± 2 °C for 6 months. Isolation of fungi from the compost was conducted using the method of enriched cultures. The cultures were carried out under shaken conditions (130 rpm min^−1^, 30 °C), using a liquid medium according to Lopez et al. (2006), with 0.25 % glucose and 0.2 % post-industrial lignin inoculated with 2.5 g of the compost (Korniłłowicz-Kowalska and Rybczyńska 2014b).

### Strain Selection

Research conducted in this study involved the three most effective strains of the total of 610 strains selected in the decolorization test of agarized 0.06 % Alizarin Blue Black B. All of the microfungal strains, i.e., *Haematonectria haematococca* BwIII43 (from soil), K37 (from compost) and *Trichoderma harzianum* BsIII33 (from soil), were characterized by more than 70 % color removal efficiency on agarized medium containing 0.06 % Alizarin Blue Black B after 14 days of cultivation. Preliminary studies on anthraquinone dye and post-industrial lignin decolorization in liquid cultures indicated that microscopic fungi can be very effective decolorizers (Korniłłowicz-Kowalska and Rybczyńska 2014b).

### Molecular Identification of Selected Microfungi

Taxonomic verification of the three strains of microscopic fungi was carried out using polymerase chain reaction (PCR) and nucleotide sequencing of the ITS1-5.8S–ITS2 spacer regions of the rRNA gene.

### DNA Extraction, PCR Amplification, and Sequencing

Molecular identification of selected strains was conducted using PCR and nucleotide sequencing. Genomic DNA was extracted from fresh mycelium of *H. haematococca* BwIII43 and K37 and *T. harzianum* BsIII33 by the use of Plant DNeasy Extraction Kit (Qiagen, Inc. Valencia, California). Then, 3′ fragment of 18S gene, the region of intervening ITS1 and ITS2 sequences, a fragment of 5.8S gene lying between the ITS spacers, and 5′ region of 28S gene were amplified using the primers ITS1 (5′-TCC GTA GGT GAA CCT TGC GG-3′) and ITS4 (5′-TTC CTC CGC TTA TTA ATA TGC-3′) (White et al. 1990). The PCR reaction was prepared in a sample volume of 25 μl with an addition of 0.1–1 μg of template DNA, 0.2 mM each primer, 0.2 mM deoxynucleoside triphosphates (dNPTs), 1.5 mM MgCl_2_, 1× PCR buffer, and 2.5 U Taq DNA polymerase (Qiagen). Program of the amplification reaction consisted of initial denaturation at 95 °C (5 min) and 30 cycles and denaturation at 94 °C (1 min), primer annealing at 55 °C (1 min), elongation at 72 °C (2 min), and a final elongation at 72 °C (10 min). PCR reaction was conducted in the MasterCycler personal thermal cycler (Eppendorf). The products of the amplification were separated electrophoretically in 1 % agarose gel with an addition of 10 μg ml^−1^ ethidium bromide in 1× TAE buffer (40 mM Tris/acetate, 2 mM EDTA, pH 8) and run at 8 V/cm of the gel. Single bands were observed in all reactions. Control reactions lacking DNA template gave no amplification products. The DNA bands were subsequently excised and eluted using a QIAEX II Gel Extraction Kit (Qiagen). Cycle-sequencing reactions with purified PCR products were performed on an ABI 3730 sequencing system (Applied Biosystems) using an ABI PRISM BigDye v3.1 Terminator Cycle Sequencing Ready Reaction Kit and AmpliTaq DNA polymerase according to the manufacturer’s instructions.

### Sequence Analysis

The sequences obtained were aligned using CLUSTAL X program (Thompson et al. 1994) and compared with the sequences in the GenBank database (NCBI) using the BLASTN program and a blastn algorithm version 2.2.29+ (Zhang et al. 2007; Morgulis et al. 2008).

### Culture Conditions

The experiments were conducted in static cultures in 100-cm^3^ Erlenmeyer flasks containing 50 cm^3^ of liquid Park and Robinson (1969) medium (0.2 KH_2_PO_4_, 0.1 NH_4_NO_3_, 0.5 MgSO_4_·7H_2_O g dm^−3^) with 0.25 % glucose and monoanthraquinone dyes: 0.01 % Carminic Acid, 0.03 % Alizarin Blue Black B and polyanthraquinone Poly R-478, and 0.2 % post-industrial lignin. The control treatment consisted of cultures without any addition of dye substrates and a medium not inoculated with fungi (Korniłłowicz-Kowalska and Rybczyńska 2014b).

### Evaluation of Decolorization Ability

The decolorization activity of the selected strains was measured spectrophotometrically by periodic measurements of absorbance of clear post-culturing fluids, after determining the maxima of absorbance: for Carminic Acid at *A*_495 nm_, Alizarin Blue Black B at *A*_526 nm_, Poly R-478 at 519 _nm_, and for the post-industrial lignin at *A*_430 nm_. Non-inoculated substrate served as control. The degree of decolorization of the post-culture medium was determined acc. to the formula of Lopez et al. (2006).

### Enzyme Activity Assays

The activity of horseradish-type peroxidase (HRP-like) was assayed according to the method of Maehly and Chance (1954) modified by Malarczyk (1984), using 0.01 % *o*-dianisidine (*ε*_460 nm_ = 11.3 M^−1^ cm^−1^) as the substrate in 0.1 M acetate buffer, pH 5.5, in the presence of 0.1 mM H_2_O_2_. The activity of manganese-dependent peroxidase (MnP) was determined through the oxidation of 1 mM MnSO_4_ in 50 mM sodium malonate, pH 4.5, in the presence of 0.2 mM H_2_O_2_, and subsequent determination of the Mn^+3^–malonic acid complex (*ε*_270 nm_ = 11.59 M^−1^ cm^−1^), according to the method described by Wariishi et al. (1992). The activity of lignin peroxidase (LiP) was assayed as described by Tien and Kirk (1988) using 20 mM veratryl alcohol (*ε*_310 nm_ = 9.3 M^−1^ cm^−1^) in 40 mM tartrate buffer, pH 3, in the presence of 0.4 mM H_2_O_2_. The activity of laccase (Lac) was measured according to a protocol by Leonowicz and Grzywnowicz (1981) using syringaldazine (*ε*_525 nm_ = 6.5 M^−1^ cm^−1^) as the substrate, in 0.1 M citrate-phosphate buffer, pH 5.

The adopted unit of enzymatic activity for all the enzymes studied was the specific activity (mU mg^−1^) of the protein. In all assays, one milliunit of specific enzyme activity (mU mg^−1^) was defined as the amount of enzyme that oxidized 1 μmol of substrate per minute under defined condition. The specific enzyme activity was measured at 1 μmol mg^−1^ of the protein. The protein concentration was determined by the Lowry method using bovine serum albumin as a protein standard (Lowry et al. 1954).

### Statistical Analysis

Input data were standardized to determine the main factors (enzymes) essential for the decolorization of dye substrates by strains *H. haematococca* BwIII43 and K37 and *T. harzianum* BsIII33. Next, the PCA method was applied using the normalized VARIMAX (STATISTICA v10.0, StatSoft Poland). Kaiser criterion was used to select the principal components determining decolorization abilities of the strains tested (eigenvalue component of >1). The contribution of the variables in the selected factors was determined on the basis of their factorial charge (>70), i.e., correlation coefficients between the input variables and the main components. The sum of the squares of the coefficients of principal components (factor loadings) was used to evaluate the degree of transfer of information resources contained in the input variables (the variance) by the main components selected for further analysis.

## Results

### Taxonomy and Molecular Identification

The tested strains of microscopic fungi were identified on the basis of phenotypic properties (micro- and macromorphological) as *H. haematococca* BwIII43 and K37 and *T. harzianum* BSIII33 (Korniłłowicz-Kowalska and Rybczyńska 2014b). These strains were verified with 100 and 99 % identity by genotyping using PCR and nucleotide sequencing. The PCR reaction was carried out, which amplified in a single product the partial fragments of 18S and 28S rRNA genes, non-coding ITS1, ITS2 spacers, and 5.8S rRNA gene lying between them. The products obtained for *H. haematococca* BwIII43 and K37 and *T. harzianum* BSIII33 (567, 566, and 596 bp, respectively) were subsequently sequenced. Common fragments of the sequences 3′ 18S, ITS1, 5.8S, ITS2, and 28S′5 (550, 534, and 587 bp, respectively) were compared with the sequences in the GenBank database (NCBI).

### Decolorization Ability

**Fig. 2:**
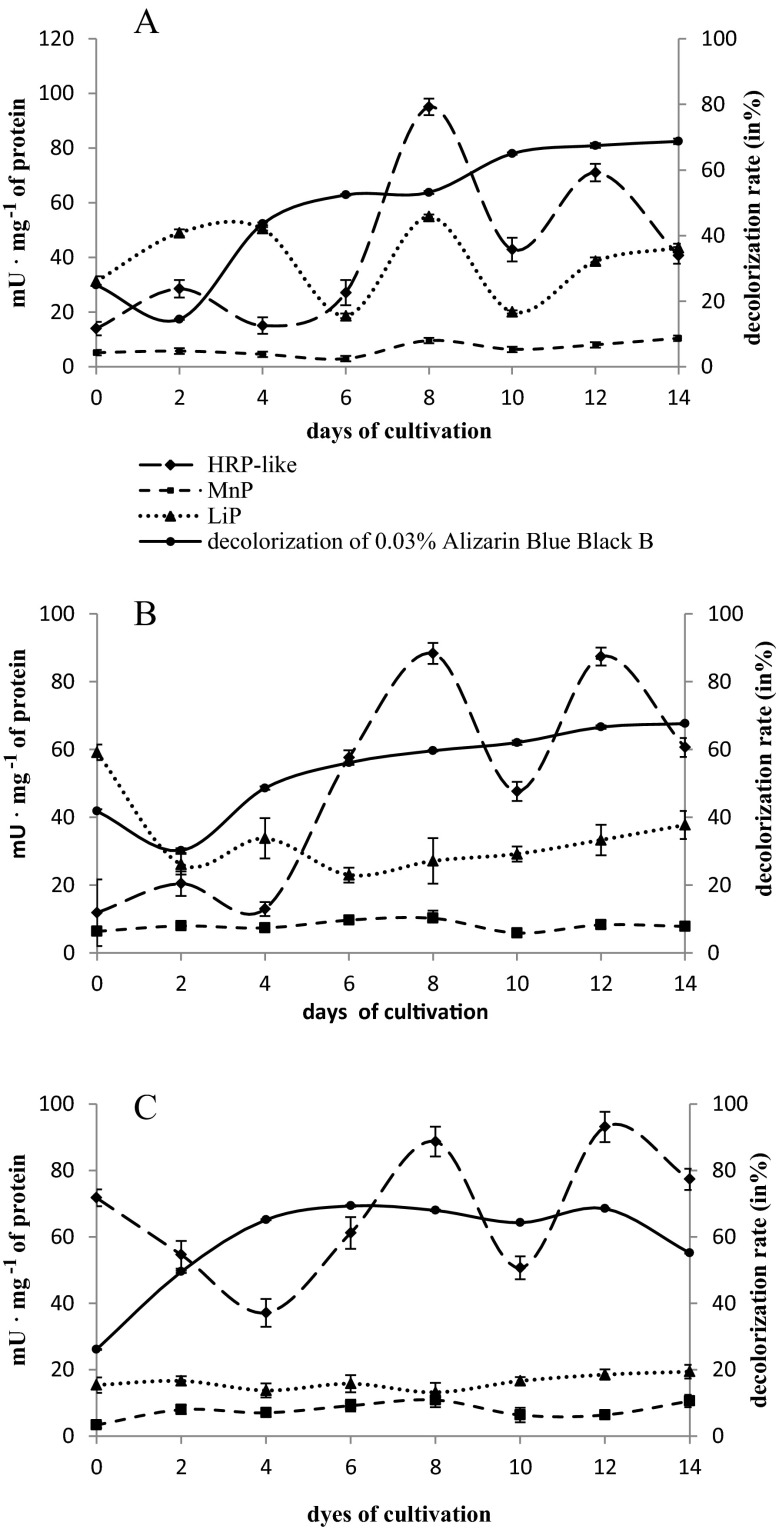
Decolorization of 0.03 % Alizarin Blue Black B and extracellular peroxidases’ activity in liquid cultures of *H. haematococca* BwIII43 (**a**) and K37 (**b**) and *T. harzianum* BsIII33 (**c**)

**Fig. 3:**
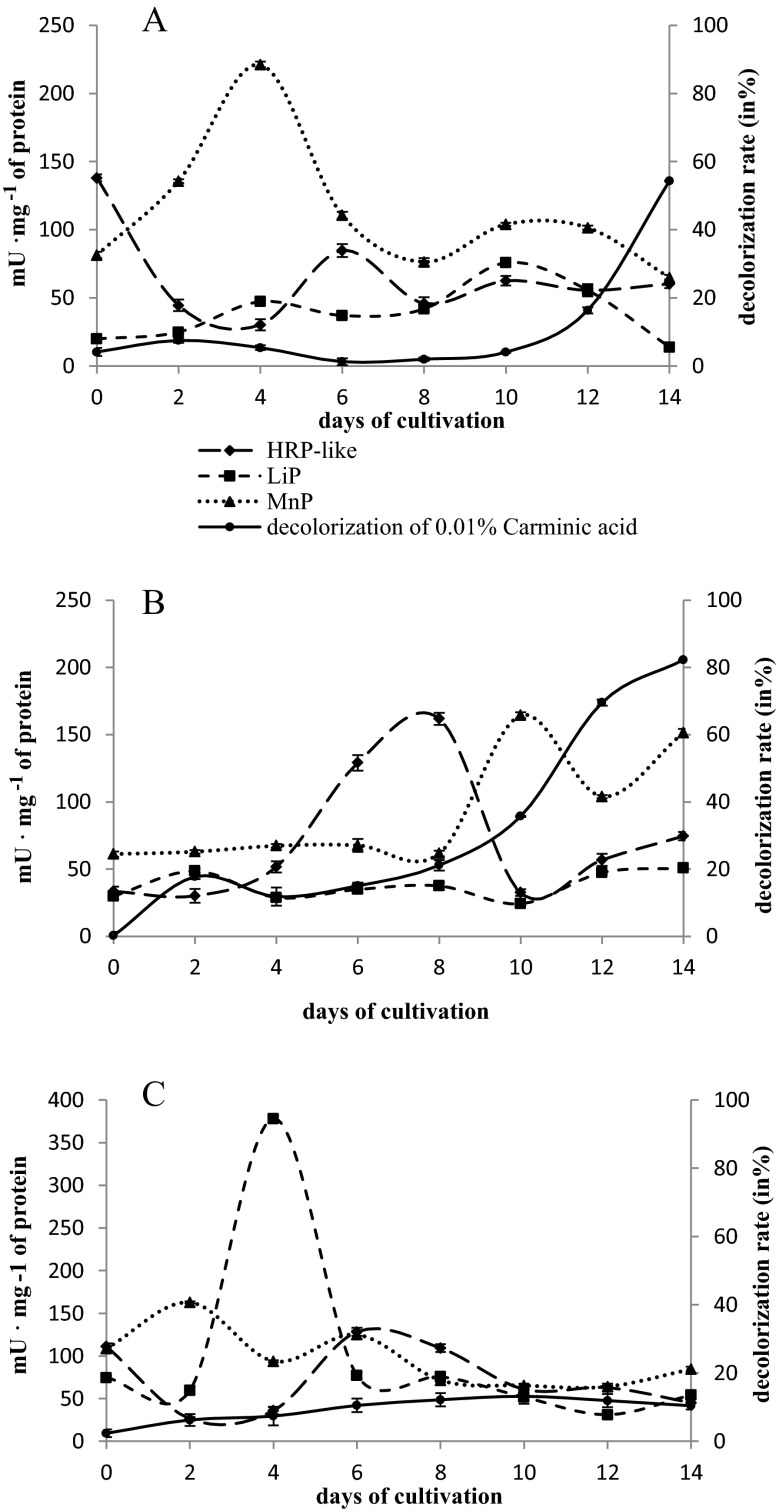
Decolorization of 0.01 % Carminic Acid and extracellular peroxidases’ activity in liquid cultures of *H. haematococca* BwIII43 (**a**) and K37 (**b**) and *T. harzianum* BsIII33 (**c**)

The tested strains of microscopic fungi—*H. haematococca* BwIII43 and K37 and *T. harzianum* BsIII33—decolorized all the dye substrates examined. The efficiency of microscopic fungi in decolorization of the individual compounds was dependent on their structure, concentration, and the strain of the fungus. *H. haematococca* strains demonstrated more effective decolorization of the dye substrates studied, particularly of monoanthraquinone dyes: 0.03 % Alizarin Blue Black B and 0.01 % Carminic Acid (Figs. 2 and 3). The strain of *T. harzianum* BsIII33 was characterized by a wide spectrum of decolorization properties with the highest affinity towards 0.03 % Alizarin Blue Black B. All the microscopic fungus strains tested efficiently decolorized 0.03 % Alizarin Blue Black B. Decolorization was most intensive in the first week of culture. Strains of *H. haematococca* BwIII43 and K37 removed 40–50 % of the color after 4 days of culturing in the presence of 0.03 % Alizarin Blue Black B, while strains of *T. harzianum* BSIII33 caused a 60 % loss of color after 2 days of culture (Fig. 2). After 2 weeks, strains of *H. haematococca* removed from 54 % (BwIII43) to 82 % (K37) of the 0.01 % Carminic Acid dye (Fig. 3).

**Fig. 4:**
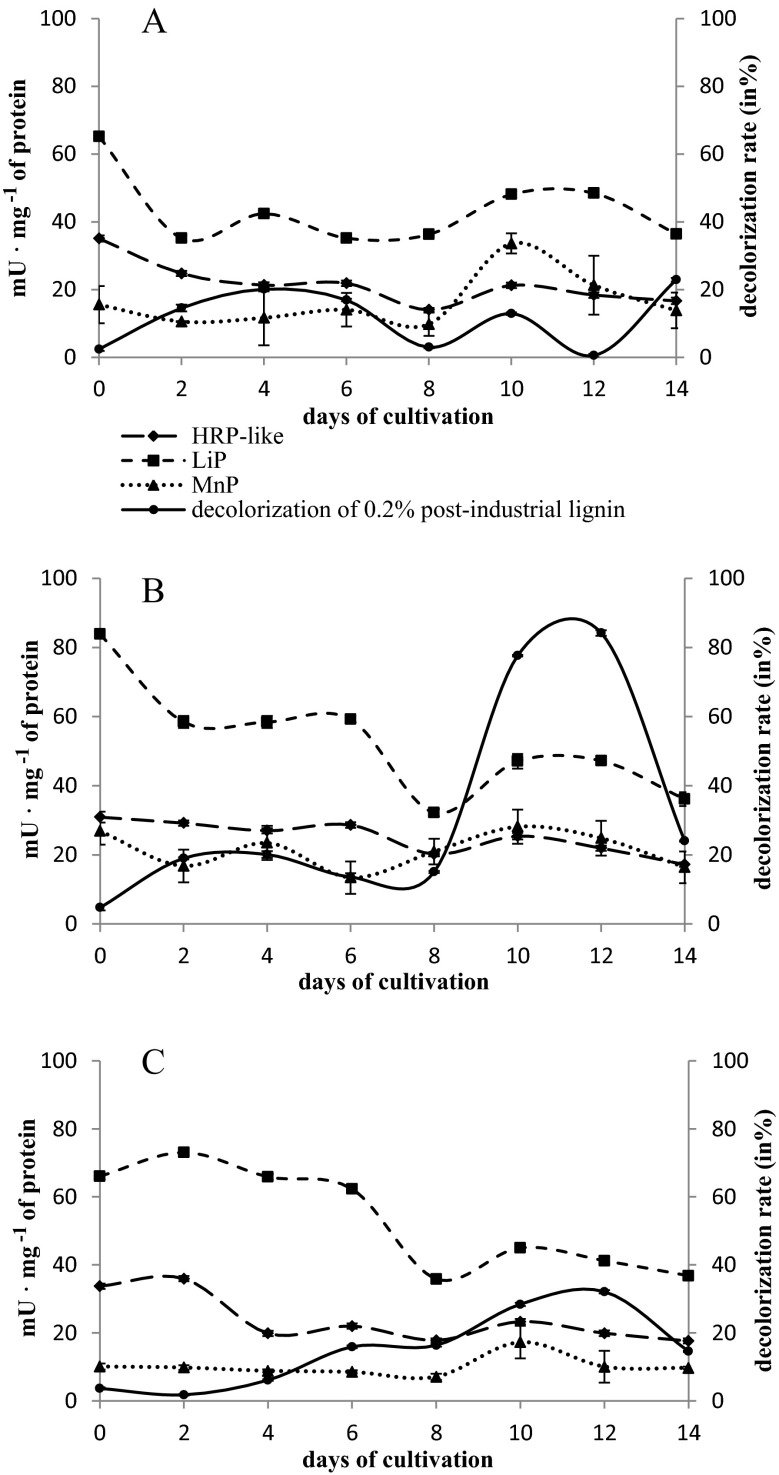
Decolorization of 0.2 % post-industrial lignin and extracellular peroxidases’ activity in liquid cultures of *H. haematococca* BwIII43 (**a**) and K37 (**b**) and *T. harzianum* BsIII33 (**c**)

The studied strains of microscopic fungi cleared markedly slower the solution of polymeric anthraquinone derivatives, 0.01 % Poly R-478 and 0.2 % post-industrial lignin. Furthermore, an increase in the intensity of the medium color was observed in the presence of these dye substrates in the second week of culture (Fig. 4 and Table 1). Only a few percent decrease in color intensity was noted on day 14 of the culture of *H. haematococca* strains BwIII43 and K37 in the presence of 0.01 % Poly R-478 substrate (2.70–4.32 %). With respect to the *T. harzianum* strain BsIII33, a systematic but low (maximum of 13.80 %) increase of dye decolorization was observed to the tenth day of culture. The highest decolorization efficiency of the post-industrial lignin was demonstrated by *H. haematococca* strain K37, which within 2 weeks of culture removed 84.20 % of the color caused by 0.2 % post-industrial lignin (Fig. 4a–c).

**Table 1:** Decolorization (in %) of 0.01 % Poly R-478 in stationary cultures of microfungi

Strain	Days of cultivation							
	0 (18 h)	2	4	6	8	10	12	14
*H. haematococca* BwIII43	0.37^a^ (±0.03) SD	2.13 (±0.03)	0.42 (±0.02)	0.53 (±0.33)	2.29 (±0.32)	2.31 (±0.25)	4.97 (±0.05)	4.32 (±0.26)
*H. haematococca* K37	1.20^a^ (±0.06)	2.18 (±0.02)	0.20 (±0.09)	0.81 (±0.02)	2.15 (±0.14)	1.16 (±0.04)	3.47 (±0.03)	2.70 (±0.19)
*T. harzianum* BsIII33	2.30^a^ (±0.04)	6.20 (±0.08)	7.57 (±0.03)	10.40 (±0.03)	11.86 (±0.02)	13.80 (±0.25)	11.90 (±0.02)	10.90 (±0.05)

*SD* standard deviation

^a^Decolorization (in %) of 0.01 % Poly R-478

### Enzyme Activity

The three selected strains of microscopic fungi exhibited three different types of peroxidase activity in the presence of all dye substrates: HRP-like MnP, LiP, and laccase activity. The biosynthesis of these enzymes was determined by the structure and concentration of the particular dyes. Laccase activity was detected only in the presence of anthraquinone dyes.

The highest activity of HRP-like peroxidase was recorded after 7 days of fungal cultures in the presence of monoanthraquinone dyes, especially in the case of 0.01 % Carminic Acid (84.71–129.06 mU mg^−1^ protein) (Fig. 3). The activity of HRP-like peroxidase increased systematically in the cultures with the addition of 0.03 % Alizarin Blue Black B, reaching a maximum value on day 12 (71.07–93.11 mU mg^−1^ protein) (Fig. 2). The activity of HRP-like peroxidase in the presence of 0.2 % post-industrial lignin was 2–2.5-fold lower with the most efficient biosynthesis of this enzyme in the first days of culture (24.75–35.91 mU mg^−1^ protein) (Fig. 4).

The increase in the activity of ligninases (LiP) was accompanied by post-industrial lignin transformations in the cultures of the fungi studied. Maximum LiP activity was measured mainly on day 1 of test strains’ cultures (66.04–83.84 mU mg^−1^ protein), when clearing of the medium with post-industrial lignin was observed (Fig. 4).

The activity of MnP in the cultures supplemented with 0.2 % post-industrial lignin was significantly lower compared to the activity of HRP-like and LiP (15–30 mU mg^−1^ protein) (Fig. 4). In cultures with the addition of anthraquinone dyes, MnP activity was dependent on the structure and concentration of the dye substrates. MnP reached the highest activity in the cultures enriched with 0.01 % Carminic Acid (60–220 mU mg protein^−1^) and 0.01 % Poly R-478 (70–400 mU mg protein^−1^) (Fig. 3).

Laccase activity was low in comparison to the peroxidase activities detected (Table 2). The highest laccase activity was detected in cultures of *H. haematococca* strains BwIII43 and K37, in the presence of 0.01 % Carminic Acid (2.0–45.0 mU mg^−1^ protein) (Tables 2 and 3).

**Table 2:** Activity of extracellular laccase in cultures of microscopic fungi in the presence anthraquinone dyes

Strains	Days of cultivation							
	0 (18 h)	2	4	6	8	10	12	14
*H. haematococca* BwIII43	10.64^a^ (±0.04) SD 0.57^b^ (±0.08) 2.46^c^ (±0.15)	5.80 (±0.38) 0.42 (±0.07) 6.77 (±1.36)	17.09 (±2.27) 0.50 (±0.04) 8.24 (±0.35)	3.94 (±0.05) 0.72 (±0.08) 7.46 (±0.40)	33.58 (±0.20) 1.29 (±0.03) 8.20 (±1.45)	4.94 (±0.09) 1.37 (±0.05) 7.01 (±1.24)	9.11 (±1.97) 0.25 (±0.09) 11.08 (±0.56)	6.16 (±1.33) 0.13 (±0.03) 4.80 (±1.35)
*H. haematococca* K37	5.08^a^ (±1.26) 1.86^b^ (±0.05) 6.78^c^ (±1.19)	2.18 (±0.17) 0.54 (±0.07) 5.97 (±3.10)	18.43 (±5.00) 0.60 (±0.05) 7.81 (±1.38)	5.60 (±1.38) 0.48 (±0.11) 8.82 (±1.56)	5.12 (±1.25) 0.92 (±0.19) 8.46 (±1.49)	29.47 (±5.40) 0.95 (±0.07) 11.90 (±2.60)	43.25 (±4.03) 0.19 (±0.03) 14.48 (±2.95)	44.98 (±1.81) 0.08 (±0.01) 8.79 (±4.14)
*T. harzianum* BsIII33	2.90^a^ (±0.19) 0.61^b^ (±0.20) 4.96^c^ (±0.13)	2.92 (±0.34) 0.31 (±0.24) 10.57 (±1.49)	16.72 (±1.59) 0.92 (±0.36) 5.97 (±2.15)	6.61 (±0.77) 0.92 (±0.12) 6.09 (±1.69)	5.93 (±1.46) 0.88 (±0.17) 6.03 (±2.09)	7.00 (±2.83) 0.90 (±0.12) 4.07 (±3.10)	3.04 (±1.31) 0.37 (±0.03) 4.30 (±2.00)	4.52 (±0.67) 0.14 (±0.01) 2.71 (±0.96)

*SD* standard deviation

Laccase activity (mU mg^−1^ of protein) in the presence of 0.01 % Carminic Acid, 0.03 % Alizarin Blue Black B, and 0.01 % Poly R-478

^a^Carminic Acid (0.01 %)

^b^Alizarin Blue Black B (0.03 %)

^c^Poly R-478 (0.01 %)

**Table 3:** Activity of extracellular peroxidases in cultures of microscopic fungi in the presence of 0.01 % Poly R-478

Strains	Days of cultivation							
	0 (18 h)	2	4	6	8	10	12	14
*H. haematococca* BwIII43	49.04^a^ (±5.34) SD 21.05^b^ (±2.97) 69.03^c^ (±0.13)	53.43 (±8.89) 29.73 (±4.15) 81.40 (±0.09)	33.71 (±5.95) 28.13 (±3.85) 77.04 (±0.05)	55.78 (±2.14) 31.03 (±6.75) 156.90 (±0.10)	66.08 (±6.67) 15.75 (±0.95) 115.00 (±0.46)	44.37 (±1.41) 13.47 (±2.12) 196.70 (±0.95)	63.71 (±0.93) 28.38 (±1.24) 155.46 (±3.46)	52.93 (±0.35) 19.63 (±3.47) 403.20 (±0.65)
*H. haematococca* K37	35.11^a^ (±5.55) 17.36^b^ (±3.07) 142.00^c^ (±0.73)	36.66 (±6.48) 15.30 (±2.90) 195.50 (±1.48)	26.97 (±1.03) 15.00 (±3.12) 82.17 (±3.95)	50.75 (±1.77) 16.94 (±2.10) 92.78 (±0.78)	48.66 (±3.88) 16.20 (±3.58) 88.95 (±2.33)	42.14 (±3.95) 9.58 (±3.31) 77.04 (±1.40)	127.71 (±7.85) 12.35 (±2.36) 101.51 (±1.14)	62.93 (±3.57) 7.50 (±1.16) 82.17 (±3.17)
*T. harzianum* BsIII33	38.06^a^ (±5.38) 10.60^b^ (±3.00) 69.58^c^ (±0.30)	97.31 (±6.88) 21.66 (±7.65) 88.95 (±0.48)	50.41 (±6.48) 102.00 (±9.53) 83.77 (±0.73)	60.75 (±6.09) 70.20 (±6.36) 112.80 (±4.05)	60.15 (±6.54) 69.51 (±6.30) 84.60 (±1.23)	40.63 (±4.42) 76.51 (±1.72) 57.14 (±3.20)	26.40 (±4.66) 42.23 (±2.60) 60.33 (±0.97)	43.76 (±5.94) 24.35 (±4.92) 57.14 (±0.75)

Activity (mU mg^−1^ of protein) of HRP-like, LiP, and MnP

*SD* standard deviation

^a^HRP-like

^b^LiP

^c^MnP

### Determination of the Main Decolorization Factor by PCA

**Fig. 5:**
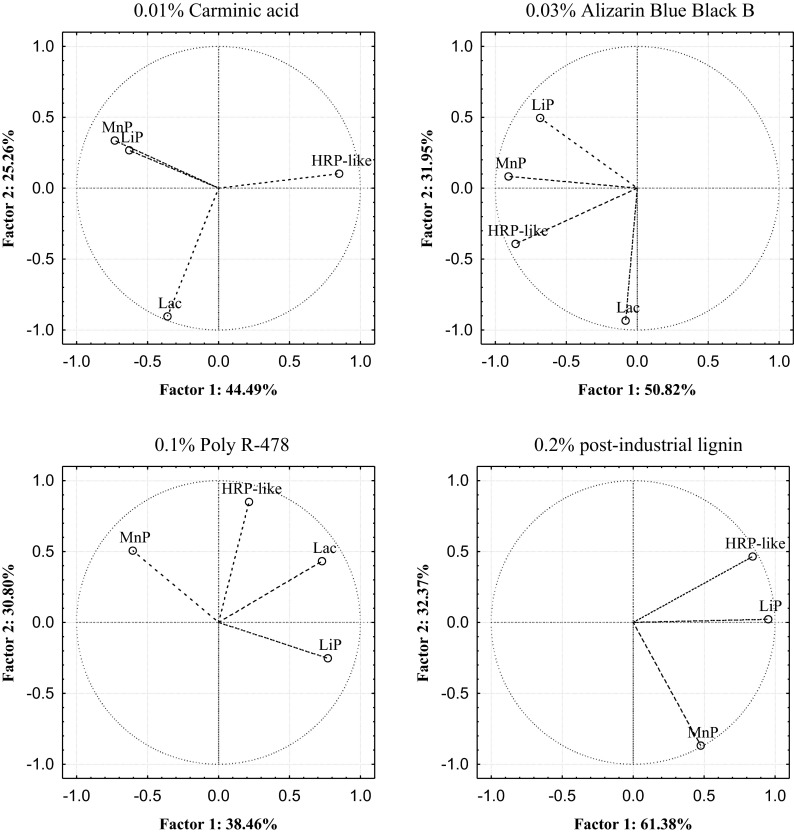
Plot of variables. Location of load vectors towards two principal components for *H. haematococca* BwIII43 strain

**Fig. 6:**
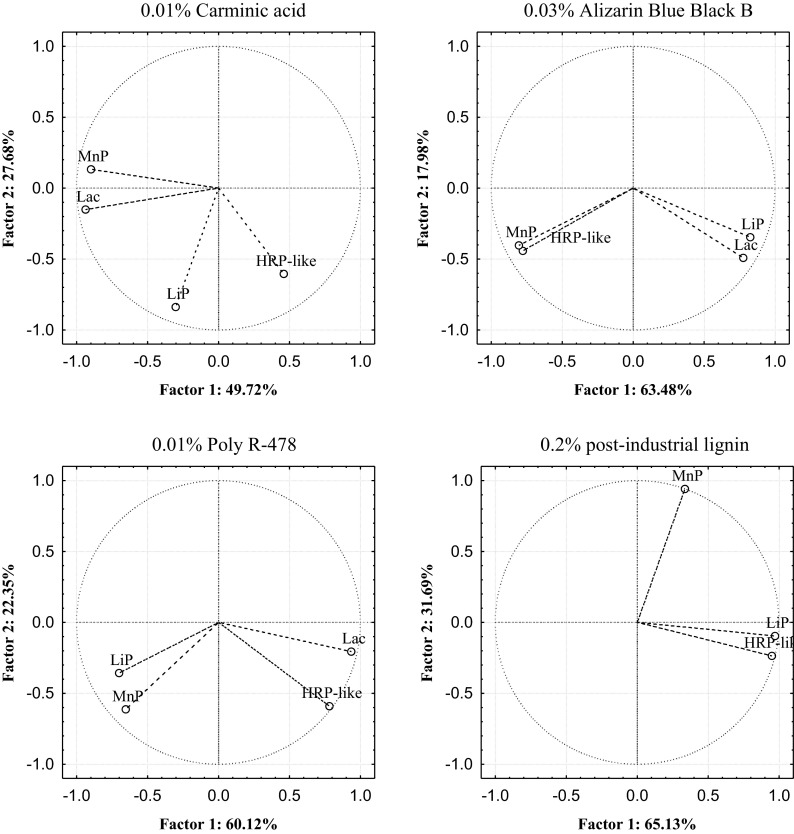
Plot of variables. Location of load vectors towards two principal components for *H. haematococca* K37 strain

**Fig. 7:**
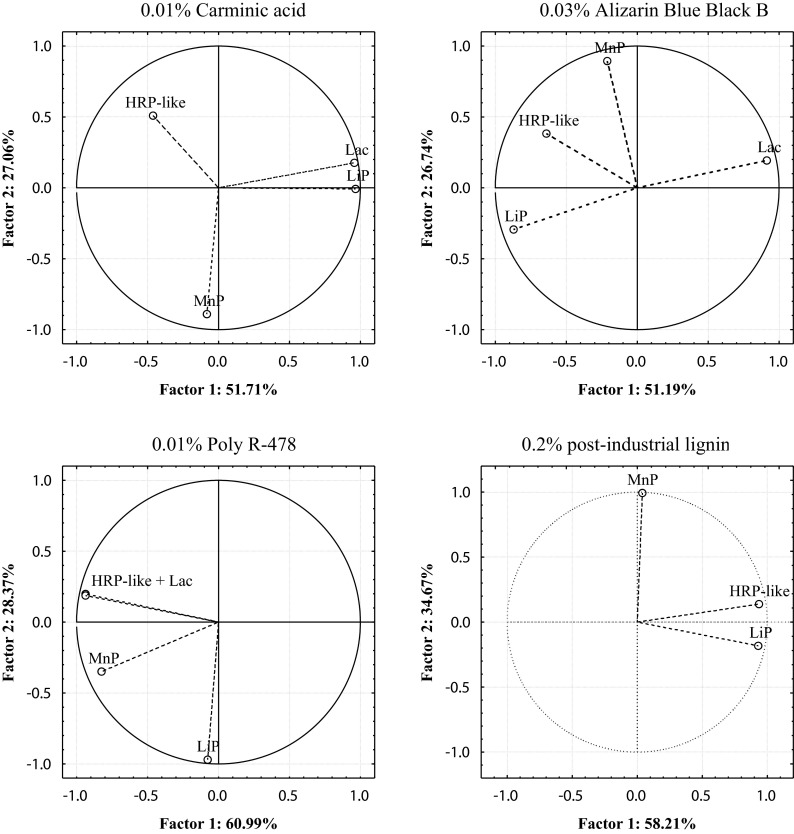
Plot of variables. Location of load vectors towards two principal components for *T. harzianum* BsIII33 strain

The main enzymes of microscopic fungi responsible for the decolorization of anthraquinone dyes and post-industrial lignin were designated on the basis of factor analysis of PCA criteria adopted. Decolorization of 0.2 % post-industrial lignin by the strains of *H. haematococca* BwIII43 and K37 and *T. harzianum* BsIII33 was strong and amounted to 58.20, 61.38, and 65.13 %, respectively, and was conditioned by the activity of HRP-like peroxidase (71–90 %) and LiP (87–94 %) (Figs. 5, 6, and 7). Participation of MnP peroxidase in the decolorization of 0.2 % post-industrial lignin by microscopic fungi tested was twofold lower (31.69–34.66 %) (Table 4). PCA found that the proportion of oxidoreductases contributing to the decolorization of anthraquinone dyes by microscopic fungi varied. In *H. haematococca* BwIII43 cultures, two factors (PC1 and PC2) were found responsible for the decolorization of monoanthraquinone dyes, Carminic Acid and Alizarin Blue Black B, which explained 69.74 and 82.77 % of the data variability, respectively (Table 4). In both cases, the first factor (PC1), associated with the activity of peroxidases, was crucial in the decolorization of these dyes and was mainly associated with positively correlated HRP-like and MnP enzyme activities. In cultures of *T. harzianum* strain BSIII33, with the addition of 0.01 % Carminic Acid and 0.03 % Alizarin Blue Black B, decolorization of these substrates occurred mainly in the presence of lignin peroxidase and laccase. Activities of these enzymes were negatively correlated. PCA in cultures supplemented with 0.01 % Poly R-478 showed the most diverse mechanism of decolorization. The percentage of the total variance determined for the first two factors (PC1 and PC2) in BwIII43 strain cultures was 38.46 and 30.79 %, respectively (Table 4). This indicated that all oxidoreductases tested were involved in the decolorization of polyanthraquinone dye. With respect to the mechanism of enzymatic decolorization of 0.01 % Poly R-478 in cultures of *H. haematococca* K37 and *T. harzianum* BsIII33, PCA method revealed that HRP-like peroxidase and laccase played a key role in these reactions (Figs. 5, 6, and 7).

**Table 4:** Eigenvalues of the decolorization parameters of selective strains

*H. haematococca* BwIII43								
Principal components	0.01 % Carminic acid		0.03 % Alizarin Blue Black B		0.01 % Poly R-478		0.2 % post-industrial lignin	
	^a^% TV	^b^% C	% TV	% C	% TV	% C	% TV	% C
1 2	44.48 25.25	44.48 69.74	50.82 31.95	50.82 82.77	38.46 30.79	38.46 69.26	61.38 32.37	61.38 93.75
*H. haematococca* K37								
1 2	49.72 27.68	49.72 77.40	63.47 17.98	63.47 81.46	61.12 22.35	61.12 82.47	65.13 31.69	65.13 96.82
*T. harzianum* BsIII33								
1 2	51.70 27.05	51.70 78.76	51.19 26.74	51.19 77.93	60.99 28.36	60.99 89.35	58.20 34.66	58.20 92.87

^a^% of total variance

^b^Cumulative %

## Discussion

The selected microscopic fungi demonstrated a similar or even higher removal percentage of anthraquinone dye solutions when compared to the white rot fungus. This particularly applied to *H. haematococca* strains, which could cause a loss of 40–50 % of the medium color stained by 0.03 % (300 mg l^−1^) Alizarin Blue Black B. For comparison, Yemendzhiev et al. (2009) showed that the strain of white rot basidiomycete, *T. versicolor* 1 in a liquid medium containing 125 mg l^−1^ of Reactive Blue 4, removed 40 % of the color after 8 days of growth. Previous studies (Korniłłowicz-Kowalska and Rybczyńska 2012) showed that the anamorphic white rot fungus *B. adusta* CCBAS 930 required a 7-day incubation period to account for the 20 % color loss during growth on liquid media containing 100 mg l^−1^ (0.01 %) of Remazol Brilliant Blue R (RBBR). The same fungus cleared 95 % of the dye only after 18 days of culture. In general, decolorization of monoanthraquinone dye solutions of 0.01 % Carminic Acid and 0.03 % Alizarin Blue Black B by fungi tested in our study was more efficient than the decolorization of monoanthraquinone dye solutions, such as 0.01 % RBBR and 0.02 % Blue 3R, in cultures of other microscopic fungi with decolorization properties (Raju et al. 2007; Anastasi et al. 2009). After 4 days, both strains of *H. haematococca* removed 40–50 % of 0.03 % Alizarin Blue Black B, while *T. harzianum* strain was able to remove 60 % of the dye after 2 days. However, as demonstrated by Raju et al. (2007), certain strains of *Fusarium* ssp. (*Fusarium oxysporum*, *Fusarium moniliforme*) and *T. harzianum* removed 36.6, 23.80, and 22.40 %, respectively, of the coloration after 4 days of culture in liquid media containing 0.02 % Blue 3R. Strains BwIII43 and K37 of *H. haematococca* and BsIII33 of *T. harzianum* showed lower capacity of 0.02 % Poly R-478 removal compared to the strains of genera *Fusarium* and *Trichoderma*, investigated by other authors (Rodriguez et al. 1996; Zheng et al.; 1999). Rodriguez et al. (1996) reported that the MUCL 35071 strain of *Fusarium solani* (= *H. haematococca*) removed more than 30 % of the color after 5 days on medium with 0.02 % Poly R-478. In the case of tested strain of *T. harzianum* BsIII33, maximum decolorization of 0.01 % Poly R-478 occurred after 10 days (13.80 %). The current study demonstrated a gradual decrease of color or a loss of coloration combined with a periodic increase in the intensity of color caused by the polymers Poly R-478 and post-industrial lignin. This may indicate a different transformation mechanism of complex aromatic compounds by different microscopic fungi species. The increase in the medium color intensity supplemented with lignin has been observed previously by Westermark and Eriksson (1974), Rodriguez et al. 1996, and Zheng et al. (1999) with respect to the white rot basidiomycete and microscopic fungi. Westermark and Eriksson (1974) reported that re-colorization of lignin was related to the formation of quinones. As a result of ligninolytic enzymes’ activity, laccases and peroxidases, colorless phenolic compounds were released from lignin and were subsequently oxidized to colored quinones (Westermark and Eriksson 1974). Re-polymerization reaction of phenols was prevented by the oxidases present in the medium (Westermark and Eriksson 1974). Zheng et al. (1999) found another explanation for the medium color intensity in cultures of decolorizing microscopic fungi. Authors concluded that the ATCC 24274 strain of *T. harzianum* caused an increase in the medium color intensity after 8 days in the presence of 0.01 % Poly R-478 by the production of extracellular pigments, masking the actual degree of decolorization. The results of our study indicate a similar mechanism of darkening of the medium with lignin and Poly R-478 in the second week of *T. harzianum* BsIII33 culture. Production of extracellular pigments was observed from the seventh day of fungal growth in cultures without the dye substrates (unpublished data). Rahouti et al. (1999) reported that the increased biosynthesis of extracellular pigments by microscopic fungi is determined by the nature of phenolic substrates used by these microorganisms. They demonstrated that the production of extracellular pigments occurred in the presence of methoxyphenols such as guaiacol, catechol, and syringic acid (Rahouti et al. 1999). This suggests that certain fungi can re-convert colorless products of biodegradation of lignin and Poly R-478 (polymers containing phenols with a methoxy group –OCH_3_) to fungal pigments.

Evaluation of decolorization activity of the fungi studied using a statistical PCA method revealed differences in the decolorization mechanism of aromatic substrates. These differences were based on various degree of involvement of peroxidases but not the laccase in the decolorization reactions of anthraquinone mono- and polymeric dyes. This finding is consistent with the results of Moreira et al. (1997) and Robinson et al. (2001) on enzymatic decolorization of aromatic compounds by basidiomycetes. These authors reported that the decolorization of various synthetic dyes and kraft lignin by the white rot fungi is related to a high activity of peroxidases and low or no activity of laccases. The lack of correlation between the induction of laccase by mutants of anamorphic white rot basidiomycete, *B. adusta* CCBAS 930, and decolorization of three different dyes by this strain, i.e., Carminic Acid, Malachite Green, and Erythrosine, has been previously reported by Korniłłowicz-Kowalska and Iglik (2007). A similar relationship was observed in this study, in the presence of all dyes tested (Alizarin Blue Black B, Carminic Acid, Poly R-478). This suggests that microscopic fungi examined in the current work also utilized peroxidases for the decolorization of dye substrates.

PCA indicated that the process of decolorization of anthraquinone dyes by the strains tested *H. haematococca* and *T. harzianum* was carried out by HRP-like, LiP, and MnP peroxidases, however, lignin decolorization only by HRP-like and LiP. Generally, the method used shows that the versatile enzyme involved in the decolorization both anthraquinone dyes and post-industrial lignin in cultures of microscopic fungi is HRP-like peroxidase. It has been shown that HRP-like peroxidase synthesized by *H. haematococca* and *T. harzianum* strains in the greatest (over 90 %) was responsible for the decolorization of the lignin and post-industrial lignin. The contribution of this enzyme in the decolorization of monoanthraquinone dyes, 0.01 % Carminic Acid and 0.03 % Alizarin Blue Black B and polyanthraquinone Poly R-478, was lower, respectively, 54–74 and 70–95 % (Figs. 5, 6, and 7). Using the PCA showed also that HRP-like peroxidase-tested micromycetes has higher affinity to anthraquinone derivatives of the structure of the polymeric (lignin, Poly R-478) than the monomeric (Carminic Acid, Alizarin Blue Black B).

The involvement of HRP-like, LiP, and MnP peroxidases in the decolorization of anthraquinone derivatives has been previously described by many authors in basidiomycetes (Jarosz-Wilkołazka et al. 2002; Belcarz et al. 2005; Korniłłowicz-Kowalska and Iglik 2007; Korniłłowicz-Kowalska et al. 2008; Korniłłowicz-Kowalska and Rybczyńska 2012). Many of them indicated HRP-like peroxidase (*o*-dianisidine as a substrate) as an enzyme responsible for the decolorization of anthraquinone dyes, alkaline lignin, and humic acids by the white and brown rot fungi. On the other hand, Paszczyński and Crawford (1995) described ligninases (LIP) as key enzymes in lignin biodegradation by certain white rot basidiomycete. In contrast, the ability of white rot fungi to synthesize MnP in the presence of aromatic compounds, including lignin and synthetic dyes, has been previously indicated by Hofrichter (2002), Shin (2004), Belcarz et al. (2005), and Korniłłowicz-Kowalska and Rybczyńska (2014a). It can be assumed that mold fungi can synthesize various peroxidases involved in the decolorization in a similar fashion as the white rot basidiomycetes. The studies show that the activity of HRP-like peroxidase in cultures of *H. haematococca* and *T. harzianum* was particularly high in the presence of monoanthraquinone dyes, especially with Carminic Acid—a dye of natural origin. However, as determined by the PCA method, participation of this enzyme in decolorization of monoanthraquinonic dyes was lower compared with the polyanthraquinonic dye.

Biosynthesis of LiP and MnP in the presence of anthraquinone dyes was determined by the concentration and chemical structure of the dyes. In the case of LiP, the highest activity was reached in cultures with 0.03 % Alizarin Blue Black B and 0.01 % Poly R-478 (Fig. 2 and Table 3). The activity of LiP in *H. haematococca* cultures supplemented with 0.03 % Alizarin Blue Black B was reversed compared to the HRP-like activity. The highest LiP activity was observed in the first 4 days of culture (31.24–59.13 mU mg^−1^ protein). MnP exhibited a high activity in cultures with Carminic Acid and Poly R-478 and low in cultures containing Alizarin Blue Black B and post-industrial lignin.

Industrial wastewater contains various dye compounds, and our study showed that the strains of microscopic fungi tested demonstrated considerable efficiency in enzymatic removal of a wide spectrum of these compounds. Therefore, we can recommend them as potential bioremediation agents for the treatment of industrial wastewater. Furthermore, these microfungi possess broad adaptive abilities in terms of growth and development in polluted waters, which would be another advantage of these organisms as bioremediation agents. Both fungi of the genus *Fusarium* (including *F. solani*) and *Trichoderma* grow and sporulate well in aquatic environment. Their spores are also wettable, which ensures ease of dissemination (Grabińska-Łoniewska et al. 2004, 2007).

## Conclusion

The study identified the main enzymes (extracellular oxidoreductases) responsible for the decolorization of anthraquinone dyes and post-industrial lignin by selected microscopic fungi. Among them, the largest share was characterized by a HRP-like peroxidase. Polymeric anthraquinone derivatives (post-industrial and lignin, Poly R-478) showed lower decolorization efficiency in comparison with monoanthraquinone dyes (Carminic Acid, Alizarin Blue Black B). However, the share of HRP-like peroxidase in decolorization of polymeric derivatives was higher than in the decolorization of monoanthraquinone dyes. Optimization of culture conditions of strains of *H. haematococca* BwIII43 and K37 and *T. harzianum* BsIII33 should result in an increased decolorization efficiency.

## Electronic Supplementary Material

ESM 1 (DOCX 22 kb)

## Abbreviations

PCA: Principal component analysis
PDA: Glucose–potato media
HRP-like: Horseradish-type peroxidase
MnP: Manganese-dependent peroxidase
LiP: Lignin peroxidase
Lac: Laccase
PCR: Polymerase chain reaction
TAE: Tris/acetate buffer
EDTA: Ethylenediaminetetraacetic acid
dNPTs: Deoxynucleoside triphosphates
