# Supplementary material for: Evaluation of Dye Compounds’ Decolorization Capacity of Selected H. haematococca and T. harzianum Strains by Principal Component Analysis (PCA)
Source: Water Air Soil Pollut. 2015 Jul 1;226(7):228. doi: 10.1007/s11270-015-2473-8 (PMC4485695; doi:10.1007/s11270-015-2473-8)
Supplement: Supplementary file 1 — (DOCX 22 kb) [file 11270_2015_2473_MOESM1_ESM.docx]

A

TGCTTAAGTTCAGCGGGTATTCCTACCTGATTCGAGGTCAACATTCAGAAGTTGGGTGTTTTACGGCATGGCCGCGCCGCTCTCCAGTTGCGAGGTGTTAGCTACTACGCAATGGAAGCTGCGGCGGGACCGCCACTGTATTTGAGGGACGGCGTGTGCCCACAGGGGGCTTCCGCCGATCCCCAACGCCAGGCCCGGGGGCCTGAGGGTTGTAATGACGCTCGAACAGGCATGCCCGCCAGAATACTGGCGGGCGCAATGTGCGTTCAAAGATTCGATGATTCACTGAATTCTGCAATTCACATTACTTATCGCATTTCGCTGCGTTCTTCATCGATGCCAGAGCCAAGAGATCCGTTGTTGAAAGTTTTAATTTATTTGCTTGTTTACTCAGAAAAACATTATAAAAACAGAGTTAGGGGTCCTCTGGCGGGGGCGGCCCGTTGTTACAGGGCCGTCTGTTCCCGCCGAAGCAACGTTTTAGGTATGTTCACAGGGTTGATGAGTTGTATAACTCGGTAATGATCCCTCCGCAGGTTCACCTACGGAAGGATCATTCCGAGTTATACAACTCAGCACCTGT

B

GCTTAAGTTCAGCGGGTATTCCTACCTGATTCGAGGTCAACATTCAGAAGTTGGGTGTTTTACGGCATGGCCGCGCCGCTCTCCAGTTGCGAGGTGTTAGCTACTACGCAATGGAAGCTGCGGCGGGACCGCCACTGTATTTGAGGGACGGCGTGTGCCCACAGGGGGCTTCCGCCGATCCCCAACGCCAGGCCCGGGGGCCTGAGGGTTGTAATGACGCTCGAACAGGCATGCCCGCCAGAATACTGGCGGGCGCAATGTGCGTTCAAAGATTCGATGATTCACTGAATTCTGCAATTCACATTACTTATCGCATTTCGCTGCGTTCTTCATCGATGCCAGAGCCAAGAGATCCGTTGTTGAAAGTTTTAATTTATTTGCTTGTTTACTCAGAAAAACATTATAAAAACAGAGTTAGGGTCCTCTGGCGGGGGCGGCCCGTTGTTACAGGGCCGTCTGTTCCCGCCGAAGCAACGTTTAGGTATGTTCACAGGGTTGATGAGTTGTATAACTCGGTAATGATCCCTCCGCA

C

GCTTAAGTTCAGCGGGTATTCCTACCTGATCCGAGGTCAACATTTCAGAAGTTGGGTGTTTAACGGCTGTGGACGCGCCGCGCTCCCGATGCGAGTGTGCAAACTACTGCGCAGGAGAGGCTGCGGCGAGACCGCCACTGTATTTCGGAGACGGCCACCCGCTAAGGGAGGGCCGATCCCCAACGCCGACCCCCCGGAGGGGTTCGAGGGTTGAAATGACGCTCGGACAGGCATGCCCGCCAGAATACTGGCGGGCGCAATGTGCGTTCAAAGATTCGATGATTCACTGAATTCTGCAATTCACATTACTTATCGCATTTCGCTGCGTTCTTCATCGATGCCAGAACCAAGAGATCCGTTGTTGAAAGTTTTGATTCATTTTCGAAACGCCTACGAGAGGCGCCGAGAAGGCTCAGATTATAAAAAAAACCCGCGAGGGGGTATACAATAAGAGTTTTAGGTTGGTCCTCCGGCGGGCGCCTTGGTCCGGGGCTGCGACGCACCCGGGGCAGAGATCCCGCCGAGGCAACAGTTTGGTAACGTTCACATTGGGTTTGGGAGTTGTAAACTCGGTAATGATCCCTCCG

Fig. 1. Sequence of nucleotide ampliefied fragments of rRNA 5.8S gene of microscopic fungi:
*H. haematococca* BwIII43 (A), K37 (B) i *T. harzianum* BsIII33 (C)

Table 1. Loads of factors (principal components)

| ***H. haemetococca* BwIII43** | | | | | | | | | |
| --- | --- | --- | --- | --- | --- | --- | --- | --- | --- |
| enzyme | | **0.01% Carminic  Acid** | | **0.03% Alizarin Blue Black B** | | **0.01% Poly  R-478** | | **0.2% post-industrial lignin** | |
|  |  | PC1* | PC2** | PC1 | PC2 | PC1 | PC2 | PC1 | PC2 |
| **HRP-like**  **LiP**  **MnP**  **Lac** | | **-0.75**  0.68  **0.80**  0.01 | 0.40  0.02  0.04  **-0.97** | **0.82**  0.69  **0.91**  0.01 | 0.45  -0.44  -0.01  **0.93** | 0.23  **-0.79**  **0.77**  -0.41 | **0.84**  0.16  0.13  **0.73** | **0.95**  **0.27**  0.08  - | 0.08  0.36  **0.98**  **-** |
| ***H. haematococca* K37** | | | | | | | | | |
| **HRP-like**  **LiP**  **MnP**  **Lac** | -0.50  0.22  **0.90**  **0.92** | | 0.56  **0.86**  -0.05  0.23 | **0.86**  **-**0.34  **0.85**  -0.20 | -0.23  **0.82**  -0.28  **0.89** | **-0.97**  0.28  0.08  **-0.83** | -0.07  **0.73**  **0.89**  -0.46 | **0.97**  **0.96**  0.08  - | 0.01  0.15  **0.99**  - |
| ***T. harzianum* BsIII33** | | | | | | | | | |
| **HRP-like**  **LiP**  **MnP**  **Lac** | 0.40  **-0.95**  0.17  **-0.97** | | -0.55  0.10  **0.87**  -0.07 | 0.49  **0.92**  -0.07  **-0.93** | -0.56  0.00  **-0.95**  0.09 | **0.95**  -0.03  **0.77**  **0.95** | -0.09  **0.97**  0.43  -0.08 | **0.93**  **0.94**  0.01  - | -0.16  0.15  **-0.99**  - |

**Explanations: *-**  principal component 1, ****** - principal component 2

Table 2. Resource values ​​of the common variation

| ***H. haemetococca* BwIII43** | | | | | | | | | |
| --- | --- | --- | --- | --- | --- | --- | --- | --- | --- |
| enzyme | **0.01% Carminic Acid** | | **0.03% Alizarin Blue Black B** | | **0.01% Poly R-478** | | **0.2% post-industrial lignin** | | |
|  | PC1* | PC1+2* | PC1 | PC1+2 | PC1 | PC1+2 | PC1 | | PC1+2 |
| **HRP-like**  **LiP**  **MnP**  **Lac** | **0.57**  0.46  **0.46**  0.00 | 0.73  0.46  0.64  **0.94** | **0.68**  0.51  **0.82**  0.00 | 0.89  0.71  0.82  **0.88** | 0.05  **0.62**  **0.60**  0.17 | **0.77**  0.65  0.62  **0.72** | **0.91**  **0.71**  0.06  - | | 0.92  0.90  **0.98**  - |
| ***H. haematococca* K37** | | | | | | | | | |
| **HRP-like**  **LiP**  **MnP**  **Lac** | 0.25  0.05  **0.82**  **0.85** | 0.57  **0.79**  0.82  0.90 | **0.74**  0.11  **0.74**  0.04 | 0.80  **0.80**  0.81  **0.84** | **0.95**  0.08  0.07  **0.70** | 0.95  **0.61**  **0.80**  0.91 | **0.95**  **0.92**  0.01  - | 0.95  0.95  **0.99**  - | |
| ***T. harzianum* BsIII33** | | | | | | | | | |
| **HRP-like**  **LiP**  **MnP**  **Lac** | 0.16  **0.92**  0.02  **0.94** | 0.47  0.93  **0.79**  0.94 | 0.24  **0.84**  0.05  **0.86** | 0.55  0.84  **0.84**  0.87 | **0.90**  0.01  **0.60**  **0.90** | 0.91  **0.94**  0.80  0.91 | **0.86**  **0.87**  0.01  - | | 0.89  0.89  **0.99**  **-** |

**Explanations: *-** resource variability principal component 1, ****** - resource variability principal component
1 and 2
